# Deciphering the Neuroautophagic Interactome: Molecular Circuits Linking Selective Autophagy to Neuropathological Cascades in Neurological Disorders

**DOI:** 10.7150/ijbs.127431

**Published:** 2026-06-10

**Authors:** Pengfei Luo, Zachary D. Travis, Cameron Lenahan, Haijian Wu, Jianmin Zhang, Jun Yu, Weilin Xu

**Affiliations:** 1 Department of Neurosurgery, Second Affiliated Hospital, School of Medicine, Zhejiang University, 88 Jiefang Rd, Hangzhou, Zhejiang 310009, China.; 2 Zhejiang Key Laboratory of Research and Transformation for Major Neurosurgical Diseases, Hangzhou 310009, China.; 3 State Key Laboratory of Transvascular Implantation Devices, Hangzhou 310009, China.; 4 Department of Medical Sciences, College of Health Sciences, Western University of Health Sciences, Pomona, Ca, USA.; 5 Burrell College of Osteopathic Medicine at New Mexico State University, Las Cruces, NM, USA.

**Keywords:** selective autophagy, neuroautophagic interactome, neurological disorders, therapeutic targets

## Abstract

Selective autophagy, a lysosome-dependent degradation pathway targeting specific substrates (e.g., mitochondria, protein aggregates), plays a pivotal role in maintaining neuronal homeostasis. Its dysregulation is intricately linked to neurodegenerative diseases, acute brain injuries, and neuroinflammatory disorders. This review elucidates the crosstalk between selective autophagy and key neuropathophysiological processes, including apoptosis, neuroinflammation, oxidative stress, and blood-brain barrier disruption. We delineate the dual roles of selective autophagy through the framework of the neuroautophagic interactome—a network in which kinases (ULK1, TBK1) and effectors (PINK1/Parkin, SQSTM1/p62) collaboratively interpret ubiquitin codes. This integrated signaling nexus functions as a decisive hub that bidirectionally modulates disease progression. Furthermore, we evaluate emerging therapeutic strategies targeting selective autophagy to mitigate neuronal damage, emphasizing its dual role as both a protector and a contributor to disease progression.

## 1. Introduction

Selective autophagy, a precise degradation system that directly maintains neuronal homeostasis by clearing dysfunctional mitochondria (mitophagy) and toxic protein aggregates (aggrephagy), governs neuronal survival and proteostasis. Additionally, it modulates apoptosis, neuroinflammation, oxidative stress, BBB integrity, and proteinopathy in neurological disorders 1,2. We propose the neuroautophagic interactome, defined as a dynamic, bidirectional regulatory network that integrates the autophagy-lysosomal machinery with the pathophysiological processes of neurological diseases. Transcending conventional linear degradation models, this framework elucidates the reciprocal interactions between molecular nodes—including autophagy receptors (e.g., p62, OPTN), ubiquitination machinery (e.g., Parkin, LUBAC), regulatory kinases (e.g., ULK1, TBK1), and functional edges in signaling and trafficking that undergo disease-specific “rewiring” 3,4. In Alzheimer's disease (AD), for instance, impaired lysosomal clearance converts autophagy into a neurotoxic driver 5, whereas in acute injuries like stroke, transient mitophagy protects penumbral neurons before transitioning to ferroptosis 6. By mapping these systemic feedback loops and decoding the interactome's molecular logic, we can identify critical hub nodes and inform stage-specific therapeutic strategies, such as nanocarrier-mediated TFEB delivery to restore proteostasis 7. Biomarkers like CSF LC3-II/p62 ratio may predict clinical outcomes 8. Here, by decoding the interactome's molecular logic, we bridge organelle-level mechanisms and circuit dysfunction, redefining autophagy as a central hub in neurological pathogenesis and therapy.

## 2. Molecular Machinery of Selective Autophagy

**Figure 1:**
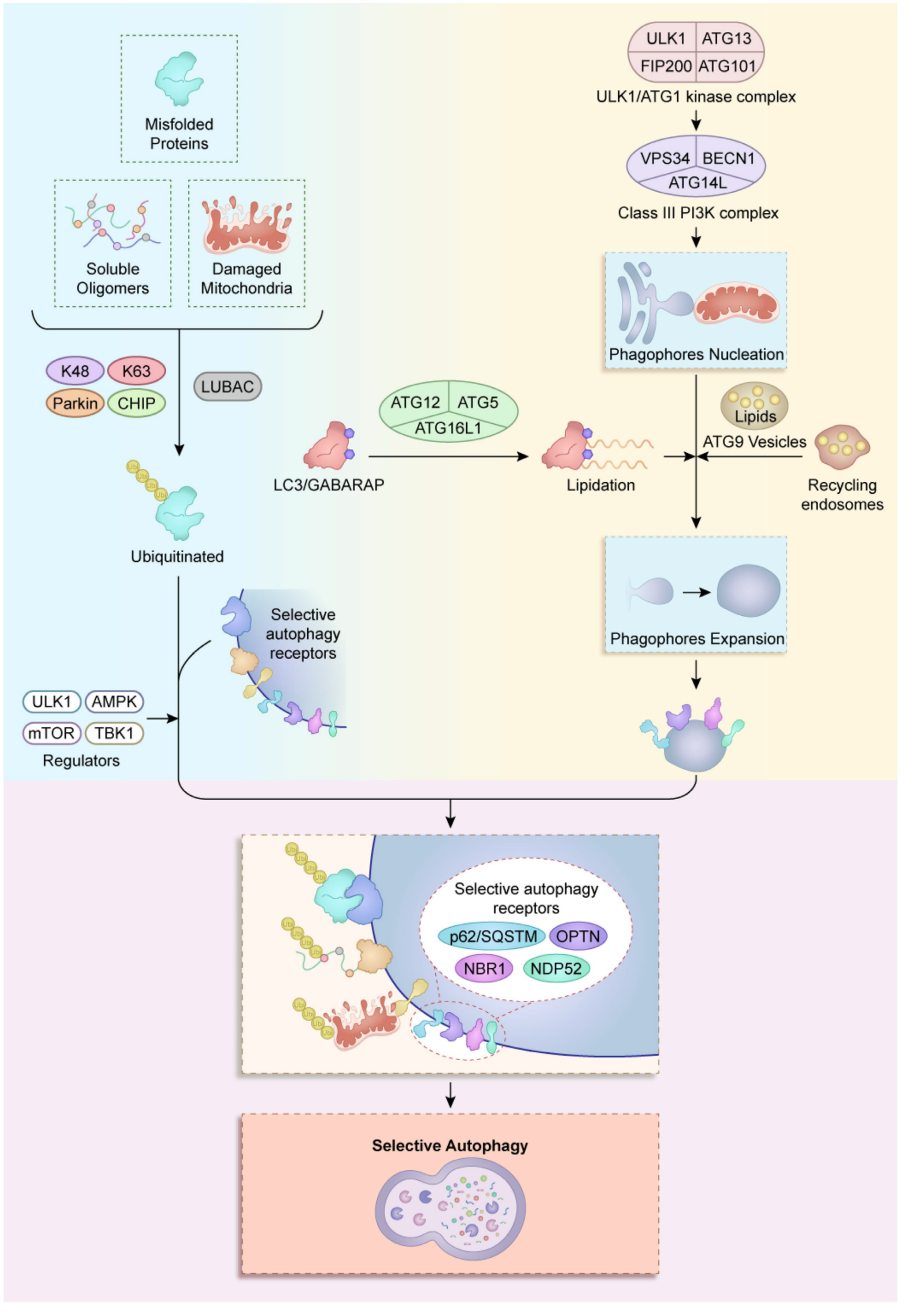
Molecular machinery of selective autophagy. Autophagy initiation is triggered by the ULK1/ATG1 kinase complex (ULK1- ATG13-FIP200-ATG101), which activates the class III PI3K complex I (VPS34-BECN1-ATG14L) to nucleate a phagophore at ER-mitochondria contact sites. Membrane expansion is then driven by two systems: (i) the ATG12-ATG5-ATG16L1 complex that promotes LC3/GABARAP lipidation, and (ii) ATG9 vesicles that deliver lipids from recycling endosomes. As the phagophore matures, selective autophagy receptors (p62/SQSTM1, OPTN, NBR1, NDP52) are recruited. Concurrently, misfolded proteins, soluble oligomers, and damaged mitochondria undergo ubiquitination through the collaboration of K48 and K63 linkages, mediated by CHIP, Parkin, and LUBAC. Ubiquitinated cargo is captured by receptor-decorated phagophores and subsequently cleared through lysosomal degradation. This process is regulated by ULK1, AMPK, mTORC1, and TBK1.

The exquisite precision of selective autophagy in neural cells arises from a tightly coordinated molecular machinery, comprising four interconnected systems: autophagy-related (ATG) proteins, cargo receptors, ubiquitination machinery, and regulatory kinases. These components collectively enable the recognition, engulfment, and degradation of specific substrates, from damaged mitochondria to pathogenic protein aggregates, while maintaining metabolic and proteostatic equilibrium in the nervous system (Figure 1).

### 2.1 Core Autophagy-Related Proteins (ATG Proteins)

Central to all autophagy pathways are the evolutionarily conserved ATG proteins, which execute autophagosome formation through hierarchical assembly. The ULK1/ATG1 kinase complex (ULK1-ATG13-FIP200-ATG101) acts as the initiation switch by integrating nutrient signals via mTORC1 and AMPK-dependent phosphorylation 9. In neurons, ULK1 exhibits compartment-specific regulation. Specifically, dendritic ULK1 is inhibited by NMDA-receptor overactivation during excitotoxicity, whereas axonal ULK1 pools are activated by local ATP depletion 10,11. Downstream of this initiation, the class III PI3K complex (VPS34-BECN1-ATG14L) catalyzes the production of phosphatidylinositol-3-phosphate (PI3P), which subsequently recruits WIPI2b and DFCP1 to nucleate phagophores at endoplasmic reticulum (ER)-mitochondria contact sites 12. Furthermore, these pathways undergo neural-specific adaptations; for instance, BECN1 plays a bifurcated role in both canonical autophagosome biogenesis and the specialized pruning of GABAergic synapses via LC3-associated endocytosis 13,14.

Membrane expansion is driven by two ubiquitin-like conjugation systems: the ATG12-ATG5-ATG16L1 complex facilitates LC3/GABARAP lipidation 15, whereas ATG9 vesicles shuttle lipids from recycling endosomes. This latter process is disrupted in Huntington's disease by mutant huntingtin binding to ATG9A's C-terminal domain 16,17. Notably, LC3 isoforms show functional divergence: GABARAPL1 preferentially binds mitochondrial prohibitin-2 during mitophagy, whereas LC3B is essential for synaptic vesicle autophagy 18. Neurons further employ unique ATG protein paralogs; for instance, cortical neurons express an ATG4B splice variant resistant to oxidative inactivation, thereby ensuring autophagic flux under high ROS conditions 19,20.

### 2.2 Selective Autophagy Receptors (p62/SQSTM1, OPTN, NBR1, NDP52)

Selective autophagy receptors serve as molecular interpreters that bridge ubiquitinated cargo to the autophagic machinery. p62/SQSTM1 oligomerizes via its N-terminal PB1 domain, clustering polyubiquitinated substrates (e.g., tau oligomers) into aggresomes, while its LIR motif recruits LC3-decorated membranes 21. In amyotrophic lateral sclerosis (ALS), mutant SOD1 disrupts p62's UBA domain, leading to defective aggregate clearance 22. OPTN (optineurin) exhibits dual mitophagy and immune functions under the regulation of TBK1 phosphorylation at Ser177. Its E478G mutation impairs both Parkin recruitment and IFN-β production in microglia 23. NBR1's J-domain enables chaperone-mediated extraction of aggregated proteins from neuronal nucleoli, while NDP52's SKICH domain targets cytosolic pathogens in astrocytes through TRIM21 collaboration 24.

Receptor redundancy and competition define neural selectivity: dopaminergic neurons prioritize OPTN for mitochondrial quality control, whereas oligodendrocytes rely on NBR1 for myelin debris clearance 25. Phase separation dynamics further regulate receptor activity as p62 undergoes liquid-liquid phase separation (LLPS) with K63-linked ubiquitin chains under oxidative stress. This process forms dynamic condensates that enhance aggrephagy efficiency 26. However, in FTLD-TDP patients, TDP-43 inclusions sequester p62's PB1 domain, which subsequently stalls condensate formation and promotes proteostatic collapse 27.

### 2.3 Ubiquitination Systems (Parkin, CHIP, LUBAC)

Ubiquitin codes dictate cargo specificity through E3 ligase-substrate pairing. Parkin, an RBR E3 ligase activated by PINK1-mediated phospho-ubiquitin signaling, tags damaged mitochondria with K63/K27 polyubiquitin chains for OPTN/NDP52 recognition. Importantly, this process requires neuronal calcium flux for full activation 28. CHIP (STUB1), in complex with HSP70, triages misfolded proteins (α-synuclein, TDP-43) to either proteasomal or autophagic degradation via substrate-specific ubiquitination: K48-linked chains target soluble oligomers to proteasomes, whereas K63 chains direct aggregates to autophagosomes 29. In Parkinson's disease, CHIP's T246M mutation biases ubiquitination toward K48 linkages, thereby exacerbating proteasomal overload 30.

The linear ubiquitin chain assembly complex (LUBAC; HOIP/HOIL-1/Sharpin) installs Met1-linked chains that stabilize autophagy-inflammation crosstalk 31. HOIP-generated linear ubiquitin on ASC specks recruits NDP52 to suppress NLRP3 inflammasomes in microglia, a checkpoint that is often disrupted in multiple sclerosis (MS) lesions 32. Paradoxically, LUBAC also enables mitophagy by linear ubiquitination of SMAC/DIABLO, preventing caspase-9 activation during mitochondrial stress 33. Such duality underscores ubiquitination's role as a rheostat balancing autophagy's cytoprotective and inflammatory outputs.

### 2.4 Regulatory Kinases (ULK1, AMPK, mTOR, TBK1)

A kinase network orchestrates autophagy dynamics across neural sub-compartments. ULK1's spatial regulation is critical: synaptic ULK1 is activated by CaMKK2 during long-term potentiation to clear aged mitochondria, while somato-dendritic ULK1 responds to AMPK-mediated glucose deprivation 34,35. AMPK itself exhibits isoform-specific roles. For instance, AMPKα2 has been reported to stabilize lysosomal V-ATPase in axons, whereas AMPKα1 promotes mitophagy via DRP1 phosphorylation 36. mTORC1's lysosomal positioning via RAGC GTPases creates subcellular autophagy mosaicism; in Alzheimer's neurons, mTOR hyperactivation at dendritic lysosomes blocks Aβ degradation but enhances Tau clearance via compartment-specific TFEB regulation 36.

TBK1 emerges as a linchpin connecting autophagy to neuroimmunity. Biochemical studies indicate that by phosphorylating OPTN (Ser177) and p62 (Ser403), TBK1 enhances their LC3-binding affinities ~20-fold, while concurrently phosphorylating STING to suppress neurotoxic type-I IFN responses 37. ALS-linked TBK1 mutations (e.g., E696K) decouple these functions by preserving OPTN phosphorylation and abolishing STING suppression, which would explain the concomitant autophagy failure and unchecked neuroinflammation observed in those cases 37. Kinase cross-talk is exemplified in ischemic stroke: HIF-1α-induced BNIP3 displaces BECN1 from BCL-2 to unleash autophagy, while the AMPK-mTOR-TBK1 axis dictates the ultimate “rebooting” capacity of the lysosomal system during reperfusion 38,39.

## 3. Selective Autophagy: A Multidimensional Interactome Bridging Cellular Stress Responses (Figure 2)

### 3.1 Selective Autophagy and Apoptosis: Molecular Switches Governing Survival and Demise

The interplay between selective autophagy and apoptosis in neurological disorders constitutes a delicate equilibrium, where molecular switches determine cellular fate. Central to this crosstalk is the Bcl-2 family/Beclin-1 axis, which serves as a rheostat balancing survival and death 40. In the context of Alzheimer's disease (AD), Aβ oligomers disrupt this axis, leading to excessive mitophagy that depletes neuronal ATP reserves 41-43. Conversely, in Parkinson's disease (PD), stabilized Bcl-2/Beclin-1 binding can suppress autophagy while simultaneously promoting BAX-mediated dopaminergic neuron apoptosis 44. This duality is exploited therapeutically; for instance, the BH3 mimetic ABT-737 disrupts Bcl-2/Beclin-1 complexes in glioblastoma, restoring autophagic flux while synergizing with temozolomide-induced apoptosis 45.

Caspase-mediated cleavage of autophagy proteins further remodels this interactome. During glutamate excitotoxicity in Huntington's disease, caspase-cleaved Bcl-2 fragments amplify mitochondrial damage 46, while caspase-8-mediated processing of SQSTM1/p62 impairs the clearance of Tau aggregates in AD neurons 47. Notably, caspase-6 exhibits paradoxical roles: its cleavage of VPS34 at Asp249 disrupts autophagosome formation in ALS motor neurons 48, while processing OPTN at Asp147 enhances OPTN's LC3 binding to sustain mitophagy in early-stage PD 49.

Beyond caspase regulation, the PINK1/Parkin axis orchestrates a mitochondrial control system. Under physiological stress, PINK1 is stabilized on depolarized mitochondria, recruiting Parkin to ubiquitinate VDAC1 with K27/K63 chains for OPTN-mediated mitophagy 50. However, sustained damage switches Parkin's activity. Specifically, in PD-linked Parkin (R275W) mutants, impaired ubiquitination leads to BAD (Bcl-2 associated agonist of cell death), opening mitochondrial permeability transition pores and triggering caspase-9 apoptosis 51. This bidirectional regulation is spatially encoded—synaptic mitochondria rely on PINK1/Parkin for routine turnover, while somatic mitochondria employ BCL2L13 for Parkin-independent mitophagy 52. Furthermore, α-synuclein mutants subvert this system by sequestering Parkin, coupling proteostatic collapse with increased apoptotic susceptibility 53,54.

In demyelinating disorders like MS, the interactome bridges ferroptosis-lipophagy crosstalk. Iron-loaded microglia undergo ferroptotic death via ACSL4-mediated lipid peroxidation, releasing oxidized phospholipids that activate astrocytic lipophagy through PPARγ-TFEB signaling 55. This response was proposed as a means to clear toxic lipid droplets but it depletes plasmalogens essential for myelin integrity. The intersection is regulated by NCOA4: during remyelination, NCOA4 mediates ferritinophagy to buffer iron, while its oxidation by 4-HNE in chronic MS redirects it to degrade lipidated LC3, stalling autophagic flux 56,57. Therapeutic modulation of this axis shows promise, liproxstatin-1, a ferroptosis inhibitor, enhances remyelination via NRF2-driven lipophagy upregulation 58.

In patients with ALS, SOD1G93A mutants induce IRE1α activation, which simultaneously cleaves Bcl-2 mRNA (reducing autophagy) and activates apoptosis 59. Moreover, ischemia-reperfusion injury induces HMGB1-dependent PARP1 hyperactivation, generating PAR polymers that block the p62-ZZ domain from binding ubiquitin, stalling aggrephagy while inducing caspase-independent apoptosis 60.

Mitochondrial injury and proteotoxicity act as decisive inputs that reshape the interactome's topology. By modulating molecular rheostats like the Bcl-2/Beclin-1 complex and PINK1/Parkin axis, these stressors force a critical transition: the interactome shifts from a homeostatic "survival mode" (promoting cargo degradation) to a "demise mode" (triggering apoptotic cascades), effectively determining the point of no return for neuronal viability.

### 3.2 Selective Autophagy and Neuroinflammation: Bidirectional Regulation of Immunity and Degradation

Neuroinflammation is a critical component in the pathogenesis of various neurological disorders, including AD, PD, MS, and ALS. It involves the activation of glial cells, particularly microglia and astrocytes, leading to the release of pro-inflammatory cytokines, chemokines, and reactive oxygen species (ROS). Selective autophagy has emerged as a key regulatory mechanism in modulating neuroinflammatory responses, offering potential therapeutic targets for these debilitating conditions 61.

The interplay between selective autophagy and neuroinflammation is complex and bidirectional. One of the most studied pathways involves the NLRP3 inflammasome, a multiprotein complex that drives the maturation of interleukin-1β (IL-1β) and IL-18. Selective autophagy negatively regulates NLRP3 inflammasome activation by degrading damaged mitochondria, a major source of mitochondrial ROS (mtROS) and mitochondrial DNA (mtDNA), which act as danger-associated molecular patterns (DAMPs) 62. In neurodegenerative diseases like AD and PD, impaired mitophagy leads to the accumulation of damaged mitochondria, perpetuating NLRP3 inflammasome activation and chronic neuroinflammation 63,64.

Moreover, selective autophagy plays a crucial role in the clearance of other DAMPs, including misfolded proteins and cellular debris, which are potent triggers of neuroinflammatory responses. For example, in ALS, the accumulation of TDP-43 aggregates activates microglia and astrocytes, exacerbating neuroinflammation 65. Autophagy pathways such as aggrephagy are essential for the degradation of these toxic protein aggregates, thereby mitigating neuroinflammatory cascades 66,67.

Parallel to proteinopathy, the STING (stimulator of interferon genes) pathway, activated by cytosolic DNA, also intersects with selective autophagy. Evidence has shown that in MS, impaired autophagy results in the accumulation of cytosolic DNA, leading to STING pathway activation and the production of type I interferons, which drive neuroinflammation and demyelination 68,69. Enhancing selective autophagy could attenuate STING-mediated neuroinflammation in MS and other neuroinflammatory disorders 69,70.

Microglial polarization, a process by which microglia switch between pro-inflammatory (M1) and anti-inflammatory (M2) phenotypes, is another interface where selective autophagy exerts control. In AD, for instance, impaired autophagy in microglia promotes a sustained M1 phenotype characterized by the release of pro-inflammatory cytokines and neurotoxic factors. Conversely, autophagy induction has been shown to promote microglial polarization toward the M2 phenotype, facilitating tissue repair and anti-inflammatory responses 71,72.

Taken together, these mechanisms illustrate how the neuroautophagic interactome is rewired in response to DAMPs and cytosolic DNA. Under inflammatory stress, the network's priority shifts from routine proteostasis to immunological regulation, where autophagic hubs serve as negative feedback loops to quench inflammasome hyperactivation and prevent a feed-forward cycle of neuroinflammation.

### 3.3 Selective Autophagy and Oxidative Stress: Redox Homeostasis Through Organelle Crosstalk

Oxidative stress is a hallmark of numerous neurological disorders, including AD, PD, ALS, and stroke. It arises from an imbalance between the production of ROS and the capacity of cellular antioxidant defenses to neutralize them 73. Excessive ROS can damage lipids, proteins, and DNA, leading to neuronal dysfunction and death. Selective autophagy, particularly mitophagy, plays a pivotal role in mitigating oxidative stress by maintaining mitochondrial quality and redox homeostasis, thus offering a potential therapeutic avenue for these diseases 74,75.

The Nrf2-Keap1-p62 pathway is a key regulatory axis that links selective autophagy to oxidative stress 76. Under conditions of oxidative stress, p62/SQSTM1 (a selective autophagy receptor) accumulates and competitively binds to Keap1, freeing Nrf2 to translocate into the nucleus. Nrf2 then activates the transcription of antioxidant genes such as HO-1 and NQO1, bolstering cellular defenses against ROS 77. In neurodegenerative disorders like AD and PD, impaired autophagy leads to the accumulation of damaged proteins and organelles, further exacerbating oxidative stress 78. Enhancing selective autophagy, particularly through the p62-Nrf2 axis, could thus bolster antioxidant responses and mitigate neuronal damage 79.

Mitophagy, the selective degradation of damaged mitochondria, is critical for reducing oxidative stress. Dysfunctional mitochondria are a major source of ROS, and their accumulation is a common feature in neurological diseases 80. For instance, in PD, mutations in PINK1 and Parkin disrupt mitophagy, leading to the accumulation of defective mitochondria and increased ROS production 81. Similarly, in ALS, impaired mitophagy contributes to mitochondrial dysfunction and oxidative stress, driving motor neuron degeneration 82,83. Therapeutic strategies aimed at enhancing mitophagy could therefore alleviate oxidative stress and improve neuronal survival in these disorders.

ROS also directly regulate autophagy at the molecular level. For instance, evidence suggests that ROS may modulate the activity of ATG4, a cysteine protease essential for processing LC3 (a key autophagy protein). Oxidative modifications of ATG4 appear to either inhibit or activate its function, depending on the extent and context of ROS exposure 84. This regulatory mechanism ensures that autophagy is appropriately tuned to the cellular redox state, preventing excessive or insufficient autophagic activity. In stroke, where oxidative stress is a major driver of neuronal injury, fine-tuning autophagy through AMPK modulation could offer protection against ischemia-reperfusion damage 85.

The thioredoxin system, which includes thioredoxin (Trx) and thioredoxin reductase (TrxR), is another critical component of the cellular antioxidant machinery that interacts with selective autophagy 86. Trx regulates redox-sensitive proteins, including those involved in autophagy, and its activity is often compromised in neurological diseases 87,88. In ALS, for example, mutations in SOD1 disrupt the thioredoxin system, leading to increased oxidative stress and impaired autophagy 89,90. Enhancing the thioredoxin system or targeting its interplay with selective autophagy could thus provide therapeutic benefits in ALS and other oxidative stress-related disorders.

Oxidative stress fundamentally reshapes the neuroautophagic interactome by focusing its activity on redox-sensitive molecular hubs. Specifically, the ROS-induced activation of the Nrf2-Keap1-p62 axis and the fine-tuning of ATG4 activity redirect the autophagic machinery toward mitochondrial quality control and antioxidant defense, establishing a defensive "redox-buffering" signature within the network.

### 3.4 Selective Autophagy and Blood-Brain Barrier Dysfunction: Dynamic Remodeling of Neurovascular Integrity

Within this complex pathological landscape, the blood-brain barrier (BBB), composed of specialized endothelial cells, pericytes, astrocytes, and tight junction proteins, serves as a dynamic interface regulating molecular exchange between the systemic circulation and the central nervous system (CNS). Emerging evidence demonstrates that the BBB is dynamically regulated by selective autophagy in endothelial cells and astrocytes 91-93. Under physiological conditions, dysfunctional or misfolded tight junction proteins (e.g., claudin-5, ZO-1) are selectively targeted for lysosomal degradation via p62-mediated aggrephagy, preventing paracellular leakage (maintaining BBB integrity) 94. However, this homeostatic process is subverted during acute injury. In ischemic stroke, evidence suggests that hypoxia-inducible factor-1α (HIF-1α) upregulates BNIP3, which triggers excessive autophagy that degrades occludin and exacerbates BBB disruption 92,95. Concurrently, damaged endothelial mitochondria release mtDNA, activating STING-dependent neuroinflammation, while selective mitophagy fails to compensate due to Parkin downregulation 68,96-100. Astrocytic end-feet may clear pathological deposits (e.g., fibrinogen) through phagocytic activity, but chronic activation could promote protease secretion (e.g., MMP-9), contributing to basement membrane degradation 101-104.

Therapeutic strategies to protect BBB integrity have shown promise: ULK1 activators (e.g., LYN-1604) have been reported to restore autophagic flux and reduce edema, and nanoparticle-mediated TFEB delivery shows promise in enhancing lysosomal clearance of hemoglobin degradation products in subarachnoid hemorrhage 105-107.

Within the neurovascular unit, stressors such as hypoxia and metabolic failure convert the neuroautophagic interactome from a stabilizer of tight junction integrity into a mediator of barrier breakdown. This transition involves a fundamental displacement of the interactome's core nodes, illustrating how external vascular stress can subvert autophagic flux to compromise the structural stability of the blood-brain barrier.

### 3.5 Selective Autophagy and Synaptic Pathology: Quality Control at the Neural Circuit Level

Parallel to the regulatory processes at the structural barriers, synaptic dysfunction and degeneration are pivotal events in the pathogenesis of numerous neurological and psychiatric disorders, reflecting the critical role of synaptic plasticity in maintaining neural network integrity. Emerging evidence highlights synaptic pathology as an early biomarker of neurodegenerative processes, often preceding overt neuronal loss.

Synaptic integrity relies on spatially constrained selective autophagy to maintain proteostasis and mitochondrial fitness at nerve terminals 108. Synaptic vesicles and aged mitochondria are preferentially degraded via local (synaptic) autophagy, a process requiring local translation of PINK1 mRNA and dynein-mediated retrograde transport of autophagosomes 109,110. In Alzheimer's disease, tau pathology (e.g., hyperphosphorylated tau accumulation) is associated with impaired autophagy, potentially contributing to synaptic dysfunction 111-113. Similarly, in Parkinson's disease, α-synuclein aggregates disrupt autophagic clearance by promoting p62 oligomerization and lysosomal dysfunction 114,115. Emerging evidence suggests that the Arc-LC3C axis, essential for memory consolidation, electively degrades inactive AMPA receptors—a pathway subverted by protein inclusions in frontotemporal dementia 116,117. Pharmacological modulation of autophagy (e.g., exercise or rapamycin) shows therapeutic potential in preclinical models, though its precise effects on synaptic restoration require further investigation 118,119.

Collectively, synaptic stressors drive the interactome toward specialized 'synaptic signatures,' such as Arc-mediated internalization and retrograde transport, to protect circuits from early erosion.

### 3.6 Selective Autophagy and Proteinopathy: Combating Phase-Transitioned Pathogenic Aggregates

Proteinopathy, characterized by the accumulation of misfolded or aggregated proteins, is a unifying pathological feature across major neurodegenerative diseases. Selective autophagy plays a pivotal yet paradoxical role in this process, acting as both a protective clearance mechanism and a potential contributor to proteostatic collapse when dysregulated 120,121. The p62/SQSTM1 adapters facilitate sequestration of ubiquitinated aggregates into autophagosomes, a process enhanced by liquid-liquid phase separation (LLPS) 122,123. This mechanism is disrupted in ALS by SOD1G93A-induced oxidation of p62 124,125. In addition to aggrephagy, HSP70-LAMP2A-reliant Chaperone-mediated autophagy (CMA), which selectively degrades soluble oligomers, is often overwhelmed in AD due to Aβ42-mediated suppression of LAMP2A transcription and ER stress-induced lysosomal dysfunction 126. HDAC6 further regulates the interactome by linking the UPS and autophagy pathways. Specifically, it recruits dynein motors to transport aggregates to the perinuclear “aggresome” for autophagic clearance—a pathway that is defective in Huntington's disease where mutant huntingtin sequesters HDAC6 127,128. Emerging therapies like ATTEC compounds (autophagosome tethering compounds), which simultaneously bind LC3 and mutant huntingtin, enhance aggregate clearance in patient-derived neurons, while TFEB activators (e.g., curcumin analogs) restore proteostasis by amplifying lysosomal biogenesis and aggrephagy capacity 129.

Chronic proteotoxicity triggers a phase-transition from liquid-like degradation hubs to solid sequestration sites. This protective isolation, when overwhelmed, leads to the total proteostatic collapse seen in neurodegeneration.

### 3.7 Selective Autophagy and Cellular Heterogeneity: Mechanisms of Pathological Convergence

While selective autophagy is a conserved homeostatic mechanism, its operational priorities are highly cell-type specific within the CNS.

Neurons exhibit a highly specialized form of "compartmentalized" clearance necessitated by their extreme polarization. Unlike most cells, neuronal autophagosomes predominantly initiate at the distal axon and must undergo retrograde transport to the soma for lysosomal fusion 130. Evidence suggests that the "dying-back" neuropathy characteristic of PD and ALS is fundamentally a failure of this long-distance transport, where the inability to maintain distal axonal mitophagy leads to synaptic collapse long before somatic death 131,132.

In contrast, microglial responses are uniquely defined by the intersection of autophagy and phagocytic pathways, often described as LC3-associated phagocytosis (LAP) or LC3-associated endocytosis (LANDO). Recent findings indicate that microglia prioritize the clearance of extracellular aggregates, such as Aβ, through these non-canonical pathways. When these processes are impaired—often via TREM2 or ATG5 dysfunction—microglia shift from a neuroprotective phagocytic state toward a pro-inflammatory phenotype, triggering NLRP3 inflammasome activation and the release of neurotoxic cytokines 133,134. Furthermore, autophagic impairment causes microglia to propagate proteotoxic "seeds" (e.g., Tau fragments) via exosomes to healthy neurons, effectively transitioning these cells from homeostatic scavengers into pathological vectors of disease dissemination 135.

In a distinct role, astrocytes and endothelial cells prioritize the architectural maintenance of the neurovascular unit. Current progress highlights that astrocyte autophagy is indispensable for maintaining neural homeostasis and proteostasis, offering a promising therapeutic target for mitigating neurodegenerative progression 136. However, chronic overactivation in these cells can lead to the paradoxical degradation of tight junction proteins, such as occludin and claudin-5, thereby facilitating blood-brain barrier (BBB) breakdown in conditions like stroke and multiple sclerosis 93.

## 4. Disease-Specific Mechanisms

The molecular interplay between selective autophagy and neurological disorders manifests through disease-specific mechanisms that reflect both shared principles and unique pathophysiological adaptations. Below, we detail how autophagy contributes to or mitigates pathology in several major conditions.

### 4.1 Alzheimer's Disease (AD)

In AD, pathogenesis is marked by a progressive transition from targeted metabolic maintenance toward a global failure in molecular recognition, intersecting with proteinopathy and neuroinflammation to drive neuronal decline. Accumulating evidence suggests that Aβ42 oligomers interact with autophagic receptors such as p62/SQSTM1, impairing cargo recognition and promoting autophagosome accumulation; this process is further exacerbated by ATG16L1 dysfunction, which disrupts autophagosome maturation through VAMP7 mislocalization 137-139. Emerging evidence suggests that Aβ aggregates sequester GABARAP family proteins (e.g., GABARAPL1/2), blocking autophagosome-lysosome fusion and generating autophagic vesicles enriched with phosphorylated Tau, which act as nucleation sites for neurofibrillary tangle formation. This proteostatic collapse is further exacerbated by PSEN1-mediated lysosomal acidification defects 140 and Aβ42-induced ER stress, which suppresses chaperone-mediated autophagy (CMA) and allows toxic Tau oligomers to accumulate 141. The resulting persistent proteostatic failure activates microglial NLRP3 inflammasomes, a process driven by mitochondrial ROS leakage from mitophagy-deficient neurons. This mechanism creates a feed-forward loop of neuroinflammation and amyloidogenesis 142-144. Hippocampal neurons initially prioritize mitophagy to counteract OXPHOS failure. However, the depletion of metabolic substrates like NAD+ shifts the interactome from selective mitophagy to non-selective bulk degradation, accelerating neuronal demise 145.

### 4.2 Parkinson's Disease (PD)

In PD, pathogenesis revolves around α-synuclein's dual exploitation of autophagy and apoptosis pathways. The A53T mutant α-synuclein impairs chaperone-mediated autophagy (CMA) by blocking LAMP2A-dependent lysosomal uptake, leading to intracellular α-synuclein accumulation. LRRK2 mutations disrupt ATG9A vesicle trafficking and autophagosome formation in axons, which exacerbates protein aggregation in synaptic terminals 141,146-148. Pathologic α-synuclein propagates via exosome-mediated transfer and tunneling nanotubes, facilitated by LRP1-mediated endocytosis in recipient neurons, thereby spreading Lewy pathology 149-151. Dopaminergic neurons exhibit selective vulnerability due to compartmentalized autophagy failure: while somatic aggrephagy (via p62-LC3 interactions) initially compensates, chronic axonal autophagic flux impairment leads to synaptic degeneration and "dying-back" neuropathy. Paradoxically, while early caspase-mediated signaling may temporarily enhance mitophagy, chronic PINK1/Parkin dysfunction eventually forces a transition to mitochondrial-driven apoptosis 152-156. Iron accumulation in the substantia nigra exacerbates oxidative stress through NCOA4-ferritinophagy defects, while lipid peroxidation from impaired glutathione synthesis drives ferroptosis 56,157.

### 4.3 Multiple Sclerosis (MS)

In MS, the autophagic machinery is uniquely redirected from lipid homeostasis toward the active execution of neuroinflammatory cascades and blood-brain barrier disruption. Myelin-derived lipid peroxidation products (e.g., oxidized phospholipids) impair ULK1-mediated autophagosome initiation in oligodendrocyte precursor cells, leading to defective clearance of myelin debris and the activation of senescence-associated secretory phenotype (SASP) 158,159. Microglial lipophagy initially resolves inflammation through NBR1-dependent lipid droplet breakdown and fatty acid release. However, chronic activation redirects autophagy machinery to process gasdermin D, thereby promoting NLRP3 inflammasome assembly and pyroptosis 160-164. Genetic susceptibility in MS is linked to CLEC16A variants, which disrupt mitophagy in oligodendrocyte progenitors by impairing PINK1/Parkin-mediated mitochondrial clearance. This results in iron accumulation due to defective NCOA4-ferritinophagy, triggering ferroptotic cell death 165-167. BBB breakdown is amplified through two synergistic pathways: 1 HIF-1α-driven BNIP3 overexpression induces excessive endothelial autophagy that selectively degrades tight junction proteins (e.g., occludin), and 2 astrocytic LC3-associated phagocytosis of fibrinogen deposits paradoxically activates MMP-9 secretion via TLR4-NFκB signaling, leading to basement membrane degradation 92,168-171.

### 4.4 Amyotrophic Lateral Sclerosis (ALS)

In ALS, the autophagic system enters a state of functional exhaustion where the clearance machinery itself is physically sequestered by toxic aggregates, a process intertwined with TDP-43 proteinopathy and oxidative stress 172. Cytoplasmic TDP-43 aggregates evade clearance by disrupting interactions with HSP90 to impede proteasomal degradation and by promoting their sequestration into stress granules. Loss of nuclear TDP-43 compromises cryptic splicing repression (e.g., STMN2 cryptic exon inclusion), exacerbating neuronal dysfunction 172-174. The C9orf72 repeat expansion produces toxic dipeptide repeat proteins that subvert autophagy initiation. For example, poly-GA aggregates sequester ATG13 to disrupt ULK1 complexes, while poly-PR peptides hyperactivate RAB7A via USP10-mediated deubiquitination, causing lysosomal exhaustion 175. Furthermore, mutant SOD1 triggers pro-apoptotic ER stress and reduces autophagic flux via Bcl-2 cleavage, while concurrent p62 accumulation recruits RUNX2 to silence lysosomal genes (e.g., CTSD) 176-178. This proteostatic collapse is further compounded by the mTORC1-dependent sequestration of TFEB in the cytoplasm, which cripples lysosomal biogenesis. Pharmacological mTOR activation (e.g., PA treatment) partially restores TFEB nuclear localization and mitigates autophagic stress in TDP-43-deficient models 178-180.

### 4.5 Stroke

In stroke, the neuroautophagic interactome exhibits a sharp temporal duality, where the rapid transition from a neuroprotective scavenger to a mediator of vascular injury defines its role in BBB regulation and apoptosis. During the acute phase (0-6 h post-insult), HIF-1α-induced BNIP3/NIX mitophagy clears ROS-generating mitochondria, preserving penumbral neurons 181,182. However, reperfusion may trigger Parkin overexpression, diverting autophagosomes toward pathological mitochondrial fission rather than degradation 183. Delayed autophagy enhancement, such as administering rapamycin at 12 h post-stroke, exacerbates hemorrhagic transformation through MMP-9 activation. In contrast, circadian-aligned therapy capitalizes on morning autophagy peaks to extend the treatment window 184. Failed endothelial mitophagy releases mtDNA that activates STING-dependent neuroinflammation, which is compounded by FUNDC1-mitophagy insufficiency due to Parkin downregulation 185. The narrow therapeutic window highlights the need for spatiotemporal precision in autophagy-targeted interventions. ULK1 activators (e.g., LYN-1604 analogs) restore pericyte autophagic flux, mitigating vasogenic edema through mTORC1-TFEB axis modulation in stroke. Neutrophil-mimetic nanocarriers can deliver TFEB plasmids to ischemic microglia, enhancing lysosomal biogenesis and debris clearance 186-188.

These disease-specific paradigms underscore that selective autophagy is not a monolithic process, but rather a modular toolkit co-opted in various aspects of neurodegeneration. Therapeutic success hinges on decoding each disease's unique “autophagy signature”, the spatiotemporal balance between protective substrate clearance and pathological pathway hijacking. Advances in single-cell autophagy flux assays and conformation-specific autophagy modulators (e.g., Aβ fibril-targeting LIR peptides) promise to usher in an era of precision autophagy therapeutics tailored to specific neurological disease states.

## 5. Therapeutic Strategies

Building upon the molecular framework of autophagic regulation, diverse pharmacological interventions are being developed to restore cellular homeostasis in pathological states. The following sections evaluate these strategies (Table 1).

### 5.1 Small Molecule Modulators

Autophagy-targeted therapies require a careful balance between enhancing protective clearance and avoiding pathological overactivation. Rapamycin analogs (e.g., everolimus) enhance Aβ clearance in AD by disrupting mTORC1-FKBP12 interactions, but their immunosuppressive effects limit their use in MS 189. Spermidine exhibits dual neuroprotective effects: in Parkinson's disease (PD), it activates PINK1-Parkin-mediated mitophagy to clear dysfunctional mitochondria, whereas in stroke models, it reduces oxidative stress and preserves blood-brain barrier (BBB) integrity by modulating the ASK1-p38 pathway.

These effects are independent of the previously hypothesized CLDN5 acetylation mechanism 190-192. Third-generation compounds like BL-918 show context-dependent effects; in ALS, BL-918 activates ULK1 selectively in motor neurons via ROS-sensitive prodrug conversion while sparing inhibitory interneurons 193. However, off-target effects persist. For example, mTOR inhibitors can indirectly upregulate chaperone-mediated autophagy (CMA) by stabilizing HSP90α, as observed in epilepsy models 194-196.

### 5.2 Gene Therapy

Gene therapy approaches are being explored to modulate autophagy pathways. AAV9 vectors effectively target the central nervous system (CNS) by crossing the blood-brain barrier (BBB), enabling TFEB overexpression to enhance lysosomal biogenesis and restore cathepsin activity in lysosomal storage disorders such as Batten disease 197,198. miR-124-3p reduces α-synuclein propagation by suppressing neuroinflammation via MEKK3/NF-κB pathway inhibition while simultaneously enhancing chaperone-mediated autophagy (CMA) through p62/p38 axis modulation. This dual mechanism reduces Lewy body formation and preserves dopaminergic neurons 199,200. TREM2 activation (e.g., via AL002a) facilitates myelin debris clearance in MS models; alternatively, CRISPRa-mediated ATG5 overexpression is proposed as a potential strategy to enhance this process, though it may risk overactivating the inflammasome 201-203. Exosome-based therapies utilizing mesenchymal stem cell (MSC)-derived exosomes show promise in TBI. These exosomes deliver miR-21 and other neuroprotective miRNAs across the BBB, enhancing autophagy-mediated mitochondrial clearance and reducing secondary injury. Neutrophil-mimetic nanocarriers loaded with TFEB plasmids further improve lysosomal function in deep brain regions, overcoming light penetration limitations of optogenetic systems 204,205.

### 5.3 Nanocarrier Systems

Nanocarrier-based delivery systems offer targeted approaches to modulate autophagy in neurological diseases. For example, PLGA nanoparticles functionalized with transferrin-receptor antibodies increase rapamycin accumulation in the brain in AD models 206. Polymeric nanocarriers decorated with lysosomal-targeting peptides (e.g., GALA) enable pH-sensitive release of hydroxychloroquine in Aβ-rich regions, representing a promising strategy to enhance microglial Aβ clearance and potentially alleviate lysosomal dysfunction 207-209. Neutrophil membrane-coated liposomes can deliver TFEB plasmids to ischemic brain regions (achieving ~90% targeting efficiency in stroke) 187,188. However, long-term exposure to certain nanomaterials may have adverse effects. For instance, prolonged use of silica nanoparticles may induce ataxia in animal models via mitochondrial ROS overproduction and impaired lysosomal acidification 210,211.

## 6. Conclusion and Perspectives

The intricate interplay between selective autophagy and neurological disorders underscores autophagy's dual role as both a guardian and an executioner of neuronal homeostasis. This review, through the lens of the neuroautophagic interactome, highlights how autophagy machinery dynamically interfaces with apoptosis, neuroinflammation, oxidative stress, and other pathological cascades. These multifaceted interactions reveal context-dependent mechanisms that dictate disease progression. In neurodegenerative diseases such as AD and PD, impaired lysosomal clearance or subversion of autophagic pathways by pathogenic proteins (e.g., Tau, α-synuclein) exacerbates proteostatic collapse. In acute injuries like stroke, transient activation of BNIP3/NIX-mediated mitophagy clears ROS-generating mitochondria during ischemia, but reperfusion triggers excessive mitochondrial fission via Parkin-Drp1 hyperactivation, leading to lysosomal exhaustion and STING-dependent neuroinflammation.

Therapeutic strategies—including small-molecule autophagy modulators and nanocarrier-mediated delivery systems-demonstrate considerable promise. However, they face challenges in balancing efficacy with off-target effects, as demonstrated by rapamycin's immunosuppressive limitations via FKBP12-dependent T-cell inhibition and the narrow therapeutic windows of ULK1 activators (e.g., LYN-1604 analogs) due to compensatory CMA upregulation in non-target cells. Emerging technologies such as CRISPR-engineered autophagy reporters and AI-driven flux models hold potential to refine precision autophagy therapeutics, but their translation will require resolving critical gaps in spatial and temporal specificity.

Despite recent advances, several limitations persist. Preclinical models often fail to recapitulate human-specific autophagy adaptations (such as neuron-specific ATG4B splice variants or circadian-regulated flux dynamics), thus limiting translational relevance. The dual-edged nature of autophagy modulation remains a major hurdle: chronic autophagy induction risks lysosomal burnout, whereas autophagy inhibition may inadvertently amplify proteinopathy. Interventional targets are intimately connected with the autophagy state and cellular homeostasis as disease progresses. Hub regulators such as AMPK, mTORC1, and SIRT1 influence not only autophagy but also multiple processes implicated in neurodegenerative diseases, including epigenetic regulation and energy homeostasis 212. Organelle-targeted autophagy regulation, rather than global autophagy, underpins selective autophagy-based therapeutic strategies. The selective clearance of damaged components, while preserving normal cellular metabolism, could substantially widen the therapeutic window 129. Biomarkers like CSF LC3-II/p62 ratio, though informative, lack disease specificity, complicating clinical stratification. Technical barriers further hinder progress 8. For instance, current imaging tools cannot dynamically track autophagosome-lysosome interactions in vivo, and the off-target effects of systemically administered autophagy-modulating compounds (e.g., TFEB activators) remain poorly characterized.

Looking ahead, the field must prioritize strategies that recalibrate, rather than uniformly boost or suppress—autophagic activity. This demands innovations in cell-type-specific delivery systems, conformation-sensitive therapies targeting pathogenic aggregates, and closed-loop neuromodulation platforms responsive to metabolic cues. Collaborative efforts integrating structural biology, nanotechnology, and computational modeling will be essential to decode the spatiotemporal logic of the neuroautophagic interactome. Ultimately, bridging these gaps will not only illuminate autophagy's role in neurological pathogenesis but also pave the way for therapies that restore proteostatic resilience without causing collateral damage to the brain's fragile ecosystem.

## Figures and Tables

**Figure 2:**
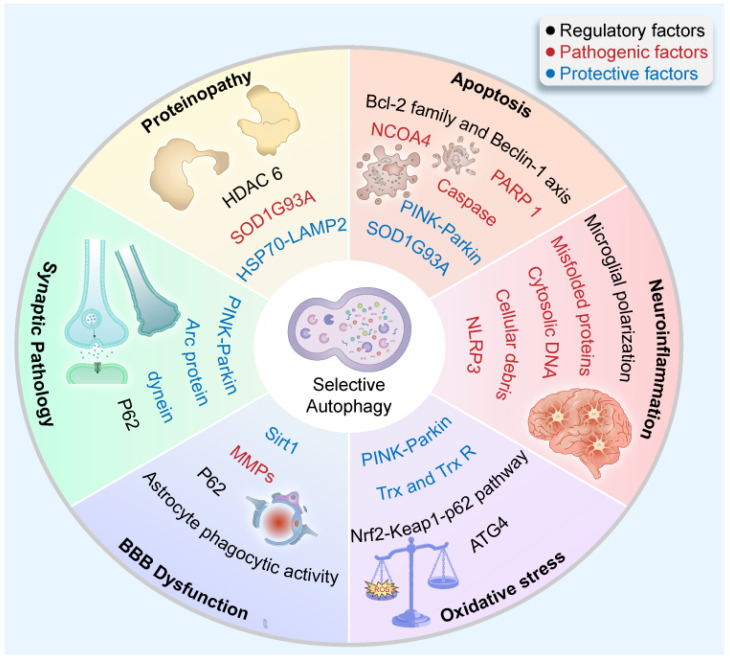
Selective Autophagy: A Multidimensional Interactome Bridging Cellular Stress Responses. Within the neuroautophagic interactome, selective autophagy exerts context-dependent effects—beneficial or detrimental—across diverse cellular stress responses in disease.

**Table 1:** Potential Therapeutic Strategies in Neuroautophagy

	Therapeutic Treatments	Disease	Molecular Target	References
**Small Molecule Modulators**	Rapamycin analogs	AD	mTORC1-FKBP12	(189)
	Spermidine	PD / stroke	PINK-Parkin / ASK p38	(190,191)
	BL-918	ALS	ULK	(193)
	mTOR inhibitors	epilepsy	HSP90α	(194,195)
**Gene Therapy**	AAV9	Batten disease	TEEB	(197,198)
	miR-124-3p	PD	MEKK3-NFκB / p62/p38 axis	(199,200)
	CRISPRa-mediated ATG5 overexpression	MS	TREM2	(201,202)
	miR-21	TBI	mitochondrial clearance	(204,205)
**Nanocarrier Systems**	PLGA nanoparticles functionalized with transferrin-receptor antibodies	AD	rapamycin accumulation	(206)
	Lysosomal-targeting peptides (eg. GALA)	AD	PH-sensitive release of choroquine	(207,208)
	Neutrophil membrane-coated liposomes with TFEB plasmids	ischemic stroke	autophagy	(187,188)

AD: Alzheimer's disease; PD: Parkinson's disease; ALS: Amyotrophic lateral sclerosis; MS: Multiple sclerosis; TBI: Traumatic brain injury

## Abbreviations

AD: Alzheimer's disease
ALS: Amyotrophic lateral sclerosis
BAD: Bcl-2 associated agonist of cell death
BBB: blood-brain barrier
CMA: Chaperone-mediated autophagy
CNS: central nervous system
DAMPs: danger-associated molecular patterns
LLPS: liquid-liquid phase separation
MS: Multiple sclerosis
MSC: mesenchymal stem cell
OPTN: optineurin
PD: Parkinson's disease
PI3P: phosphatidylinositol-3-phosphate
ROS: reactive oxygen species
TBI: Traumatic brain injury
Trx: thioredoxin
TrxR: thioredoxin reductase
UPS: ubiquitin-proteasome system
